# Effects of repetitive peripheral magnetic stimulation on spasticity evaluated with modified Ashworth scale/Ashworth scale in patients with spastic paralysis: A systematic review and meta-analysis

**DOI:** 10.3389/fneur.2022.997913

**Published:** 2022-11-08

**Authors:** Jia-Xin Pan, Ying-Xiu Diao, Hui-Yuan Peng, Xi-Zhen Wang, Lin-Rong Liao, Mao-Yuan Wang, You-Liang Wen, Yan-Bing Jia, Hao Liu

**Affiliations:** ^1^School of Rehabilitation Medicine, Weifang Medical University, Weifang, China; ^2^School of Rehabilitation Medicine, Gannan Medical University, Ganzhou, China; ^3^Department of Neurology, Zhongshan Hospital of Chinese Medicine Affiliated to Guangzhou University of Chinese Medicine, Zhongshan, China; ^4^Rehabilitation Medicine Center, The First Dongguan Affiliated Hospital, Guangdong Medical University, Dongguan, China; ^5^Department of Rehabilitation Medicine, First Affiliated Hospital of Gannan Medical University, Ganzhou, China

**Keywords:** repetitive peripheral magnetic stimulation, spasticity, systematic review, meta-analysis, rehabilitation

## Abstract

**Background:**

Spasticity is a common motor disorder resulting from upper motor neuron lesions. It has a serious influence on an individual's motor function and daily activity. Repetitive peripheral magnetic stimulation (rPMS) is a non-invasive and painless approach developed for therapeutic intervention in clinical rehabilitation. However, the effectiveness of this intervention on spasticity in patients with spastic paralysis remains uncertain.

**Objective:**

This study aimed to investigate the effectiveness of rPMS on spasticity, motor function, and activities of daily living in individuals with spastic paralysis.

**Methods:**

PubMed, PEDro, Embase, Cochrane Library, and Web of Science were searched for eligible papers with date up to March 31, 2022. Two independent researchers conducted study screening, data extraction, and methodological quality assessment. RCTs that explored the effects of rPMS on spasticity, motor function, and activities of daily living in patients with spastic paralysis were included for review. The Cochrane collaboration tool was used to assess methodological quality. The cumulative effects of available data were processed for a meta-analysis using Reedman software.

**Results:**

Eight studies with 297 participants were included. Most of the studies presented low to moderate risk of bias. Compared with the control group, the results showed that rPMS had a significant effect on spasticity (all spasticity outcomes: standardized mean difference [SMD] = −0.55, 95% confidence interval [CI]: −0.94 to −0.16, *I*^2^ = 40%, and *P* = 0.006, Modified Ashworth Scale: mean difference [MD] = −0.48, 95% CI: −0.82 to −0.14, *I*^2^ = 0%, and *P* = 0.006), motor function (Fugl–Meyer Assessment: MD = 4.17, 95% CI: 0.89 to 7.46, *I*^2^ = 28%, and *P* = 0.01), and activities of daily living (Barthel Index: MD = 5.12, 95% CI: 2.58 to 7.67, *I*^2^ = 0%, and *P* < 0.0001). No side effect was reported.

**Conclusion:**

The meta-analysis demonstrated that the evidence supported rPMS in improving spasticity especially for passive muscle properties evaluated with Modified Ashworth Scale/Ashworth Scale, as well as motor function and daily activity of living in individuals with spastic paralysis.

**Study registration:**

The reviewed protocol of this study is registered in the international prospective register of systematic reviews (PROSPERO) (CRD42022322395).

**Systematic review registration:**

https://www.crd.york.ac.uk/PROSPERO/#recordDetails, identifier CRD42022322395.

## Introduction

Spasticity is one of the common movement disorders secondary to upper motor neuron disease (UMNL). It has been defined as velocity-dependent increased muscle tone and resistance to manual stretch resulting from hyperexcitability of the stretch reflexes (1). Pandyan et al. (2) proposed that spasticity presents as involuntary activation of muscles due to UMNL. Dietz and Sinkjaer (3) reported that spasticity is often combined with a disturbance of the proprioceptive input. It is a common clinical symptom in many neurological diseases, such as cerebral palsy, multiple sclerosis (MS), stroke, traumatic brain injury (TBI), and spinal cord injury (SCI) (4).

Clinical treatment of muscle spasticity includes passive movement, stretch, active exercise, electrophysical therapy, orthotics, pharmaceutic preparation, Botulinum toxin injection (BTX), and surgery (5). Although various methods have shown certain effects on spasticity reduction, each of them has their own limitation. For example, the clinical application of BTX and pharmacological approach are limited due to the invasive method and drug side effects (6). Passive movement and stretch are routinely used in rehabilitation, but they have limited long-term effect on spasticity (7). Repetitive peripheral magnetic stimulation (rPMS) is a non-invasive and painless method with negligible side effects, which can produce a magnetic field to stimulate peripheral nervous system and muscles, and be applied to clinical practice. Previous studies have demonstrated that rPMS with single or multiple sessions can significantly reduce spasticity and increase upper limb motor function in patients following central nervous system (CNS) lesions (8–10). The underlying mechanism might be related to the neuromodulation effect when rPMS is placed over muscle or nerves of the paretic limb (9, 11–13).

In recent years, rPMS has gained popularity in neurological rehabilitation, and numerous scholars are paying attention to the effect of rPMS on spasticity related to UNML. This systematic review and meta-analysis aimed to evaluate the effects of rPMS on spasticity, motor function and activities of daily living (ADL) in patients with CNS lesion.

## Materials and methods

### Study design and registration

This systematic review and meta-analysis was conducted according to the recommendations from the Cochrane Collaboration and is reported in accordance with the Preferred Reporting Items for Systematic Reviews and Meta-analysis (PRISMA) guidelines (14). The protocol for this systematic review and meta-analysis was registered in the PROSPERO database (CRD42022322395).

### Data sources and searches

Five major databases including PubMed, Embase, Web of Science, Physiotherapy Evidence Database, and Cochrane Library were used for electronic search. Two researchers (YXD and JXP) searched for randomized controlled trials (RCTs) that met inclusion criteria from the inception of the database through March 31, 2022. To specify and limit the search scope and find topic-related studies, we used the following keywords in various combinations: “spasticity”, “spasms”, “muscular tension”, “dystonia”, “muscle hypertonia”, “repetitive peripheral magnetic stimulation”, “functional magnetic stimulation”, “peripheral magnetic stimulation”, “magnetic stimulation”, “randomized controlled trial”, and/or “controlled clinical trial”. The NCBI's Clinical Queries (15) for sensitive search strategy was used as a search filter to identify randomized trials. The detailed search strategy is showed in the Appendix. To fully identify other relevant studies, we also searched reference lists of eligible RCTs and previous reviews.

### Study selection

Articles obtained as a result of our search were imported into Endnote X9 software (Clarivate Analytics, London, UK). Here, duplicates were removed with the software. Subsequently, two reviewers screened titles and abstracts of the remaining studies. The full articles were then retrieved according to the inclusion and exclusion criteria. Any disagreements were resolved by consulting with the third reviewer (HL).

#### Populations

The studies involved adult patients suffered spasticity caused by central nervous system diseases, such as stroke, SCI, cerebral palsy, TBI, and other special conditions were included.

#### Interventions

Studies adopted interventions as rPMS alone or in combination with other rehabilitation programs except botulinum toxin. rPMS was applied to the peripheral limbs with single or multiple sessions. Furthermore, the stimulation parameters were stated in the articles.

#### Comparators

The comparators were other conventional interventions, no intervention, and sham rPMS or sham rPMS combined with other rehabilitation methods.

#### Outcomes

Studies were required to measure spasticity as the primary outcome with validated tools, such as MAS, AS, MTS. Motor function, ADL, and functional mobility were considered as secondary outcomes in the review with measurements by Fugl-Meyer Assessment (FMA), Barthel Index (BI), or other validated scales or tests. Experimental data were collected before and after treatment immediately, as well as follow-up assessment. When available, adverse events were also described.

#### Study designs

All randomized controlled trials (RCTs) including parallel and cross-over design published in English invloving rPMS for patients with spasticity were considered to be included.

### Exclusion criteria

Studies were excluded if (1) the intervention was non-peripheral and directly applied to the head, (2) non-RCTs, (3) review articles, meta-analysis, editorials, letters, comments, conference abstracts, or case reports, (4) unavailable full text, (5) above 5% participant dropped out for the primary outcome, (6) animal studies, and (7) non-English literature.

### Data extraction and management

The two authors (JXP and YXD) extracted data to a Microsoft Excel sheet manually, with discrepancies resolved through discussion. Extracted information including study characteristics (author and year of publication), demographic and clinical characteristics of the study population (total number of subjects, age, gender, clinical diagnosis, and baseline characteristics), rPMS characteristics (number of stimulation sessions, stimulation location, frequency, duty cycle, total number of magnetic pulses per session, intensity of treatment, and duration of treatment), outcome measurements, follow-up, and adverse effects. If information was missing or unable to be extracted in the process of data extraction, the corresponding author of the article was contacted by e-mail three times. If the corresponding author did not reply, we defaulted that the data information for this study could not be obtained. There was no data extracted from graphs and figures.

### Assessment of risk of bias

Two researchers (JXP and YXD) independently assessed the risk of bias for each outcome of retrieved studies according to the Cochrane Collaboration tool (RoB 2.0) involving five domains: randomization process, deviations from intended interventions, missing outcome data, measurement of the outcome and selection of the reported result (16). Each domain was deduced as low risk, high risk or some concerns by algorithm of several signal questions. Any disagreements between the two researchers were resolved by discussion with the third researcher (HL).

### Data synthesis and analysis

Meta-analysis of a given result was performed only when at least two trials used the same outcome measure. An evaluator (JXP) entered the data into RevMan software version 5.4 (Cochrane, London, UK), and another review author (YXD) checked the entries. Data analysis was based on change scores (baseline and after the last session of rPMS). For continuous data, the results were reported as standardized mean difference (SMD) and mean difference (MD) with 95% confidence interval (CI) using random effects model. Median and interquartile range will be transformed into mean and standard deviation (17). The heterogeneity of the study was assessed by *I*^2^ statistical test. Percentages of 25, 50, and 75% indicate low, medium, and high heterogeneity, respectively (18).

## Results

### Study search results

**Figure 1:**
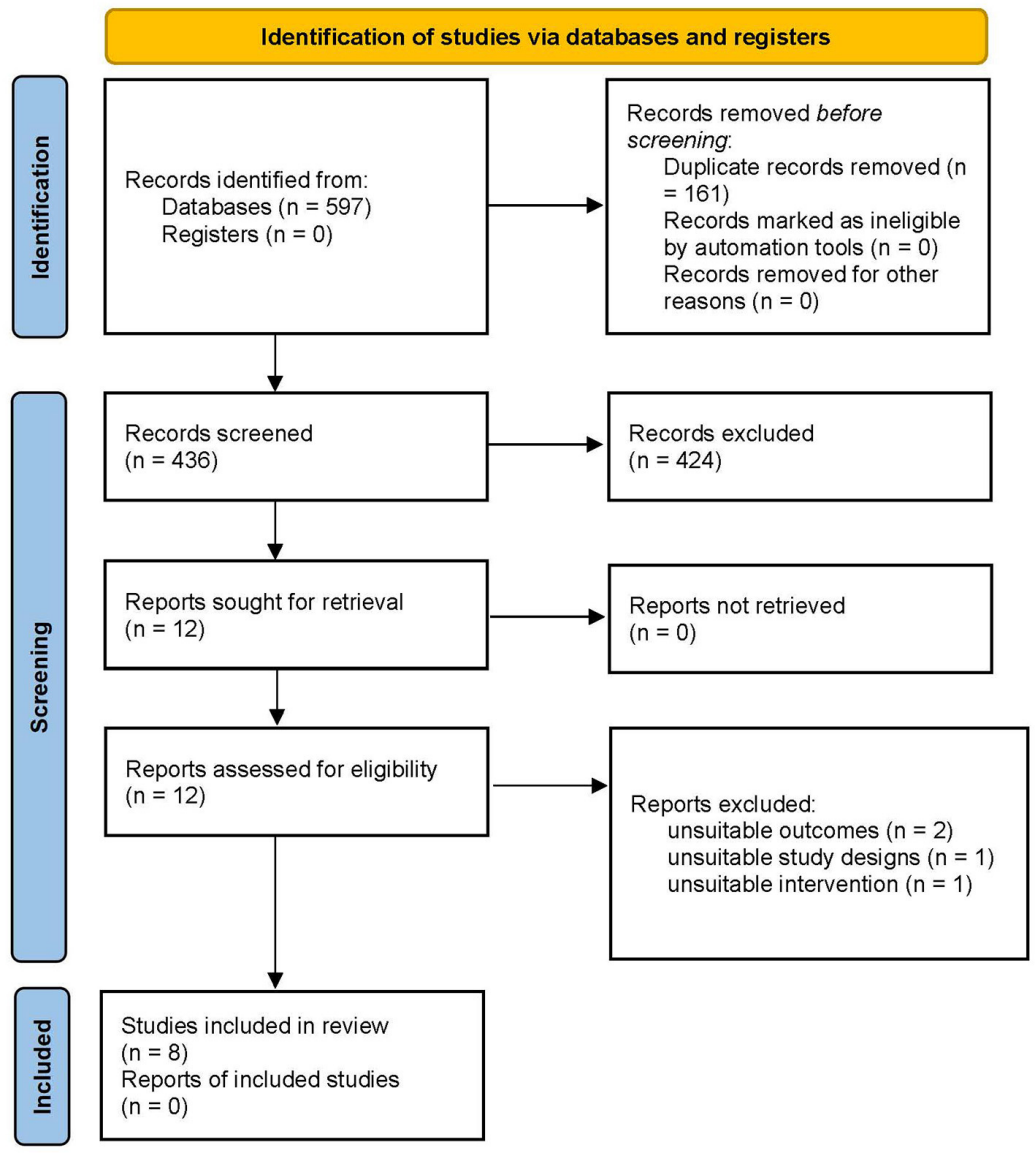
PRISMA flow diagram.

A total of 597 studies were retrieved from searches in the five major databases (Figure 1). In the 597 studies, 161 duplicates were eliminated. A total of 424 records were excluded by reading the title and abstract because they were notes and reports, meeting summaries, reviews, and meta-analyses. Twelve full-text papers were assessed for eligibility. After reviewing the full text of the 12 articles, four were excluded because of inappropriate outcome measures, study design (not RCT), and intervention (not rPMS). Ultimately, eight trials involving a total of 297 participants were included in our review. Given that the data in the two articles could not be accurately extracted (only be presented by qualitative evaluation), only six articles were included in our meta-analysis. The process of study screening is shown in Figure 1.

### Risk of bias

**Figure 2:**
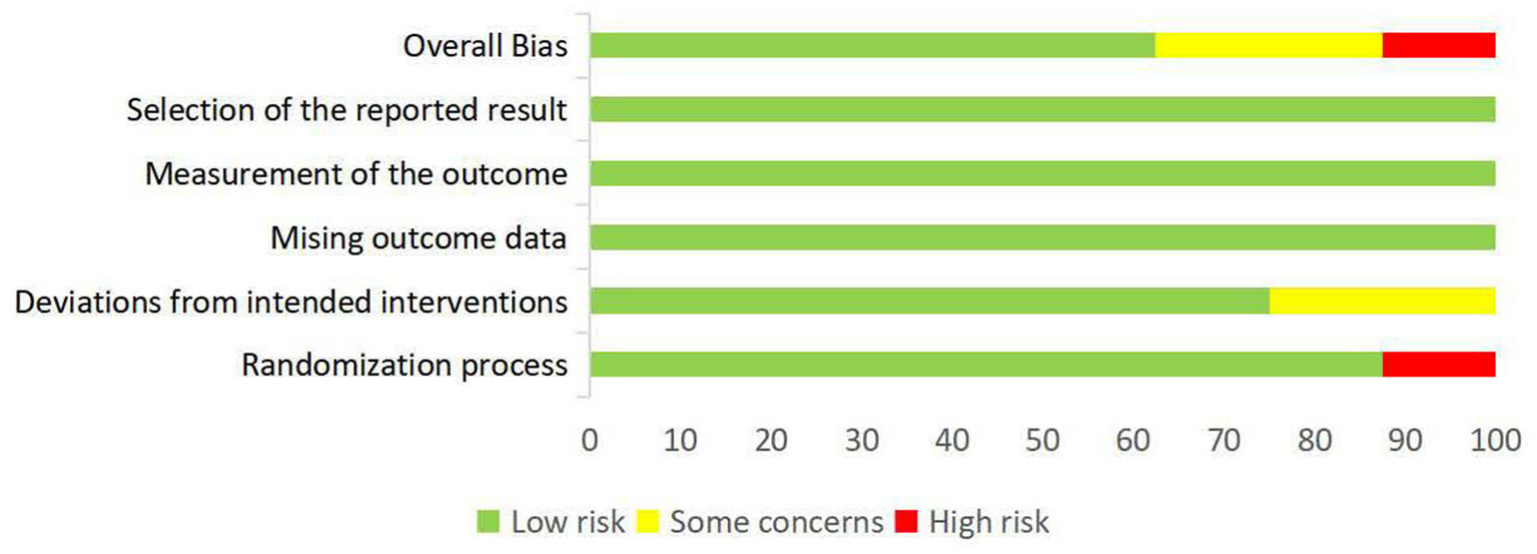
Risk of bias summary.

**Figure 3:**
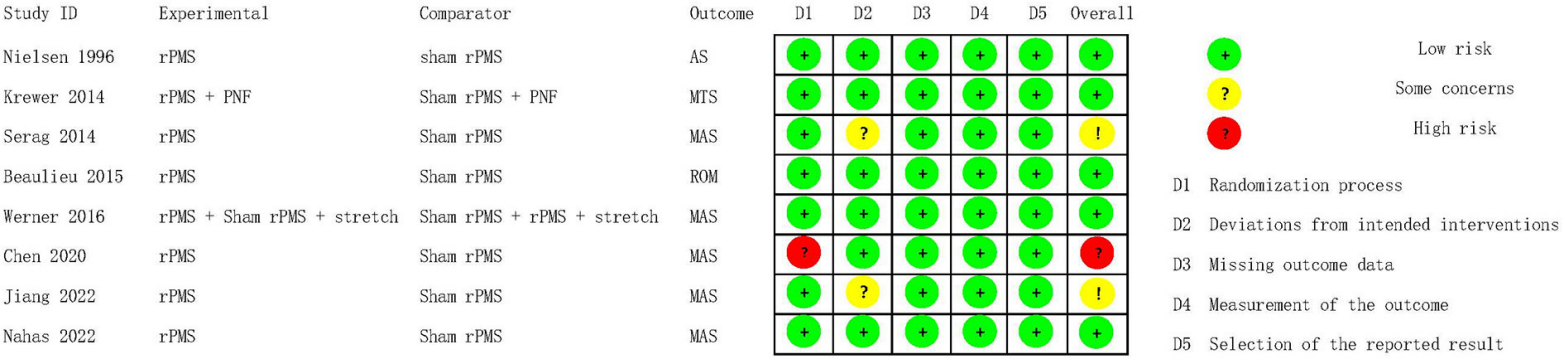
Risk of bias assessment of individual studies: no difference in the results for the outcomes assessed in each study.

The overall risk of bias of the eight included studies are summarized in Figure 2, and the assessment results of the five domains and the overall bias for individual studies are shown in Figure 3. As no difference among the results of risk of bias for each outcome assessed in per study, the main outcome of the current review was selected to present in the Figure 3. Overall, one study (19) was assessed to be of high risk of bias and two (20, 21) had some concerns, the others were at low risk of bias (22–26). Most of studies described a random sequence clearly, only one study (19) has high risk in randomization process. The potential risk of deviations from intended intervention were found in two studies (20, 21) because they did not provide clear information of blinding on the patients. No study reported that more than 5% of subjects drop-out and inappropriate outcome measures were used. Moreover, all studies showed low risk on the selection of the reported results because the reported outcome analyses were consistent with the pre-specified analysis plan. In summary, most of the studies presented low to moderate risk of bias.

### Characteristics of included studies

After a series of literature screening and qualification confirmation, eight RCTs (19–26) were selected for systematic review, and six were presented in the form of a meta-analysis. Table 1 summarizes the characteristics of the included studies and patients with spastic paralysis. The studies were published between 1996 and 2022. Table 2 summarizes the main characteristics of the rPMS parameters, and Table 3 summarizes the objectives, inclusion and exclusion criteria, results, and adverse events of all studies.

**Table 1:** Characteristics of included studies in this conducted systematic review.

**References**	**Design**	**Main diagnosis**	**Duration (mean)**	**Interventions**	**Total sample** **size**		**Age, mean (years)**	**Gender**		**Follow-up**	**Outcome measures**
					***N***	***n***		**Female**	**Male**		
Nielsen et al. (22)	RCT	MS	Group A = 12 ± 8 (years) Group B = 13 ± 7 (years)	Group A = rPMS Group B = Sham rPMS	38	Group A = 21 Group B =17	Group A = 44 ± 8.25 Group B = 44 ±10	26	12	No	AS, EMG, Self-Ease Score
Krewer et al. (23)	RCT	Stroke TBI	Group A = 26 ± 71 (weeks) Group B = 37 ± 82 (weeks)	Group A = rPMS + PNF Group B = Sham rPMS + PNF	63	Group A = 31 Group B = 32	Group A = 55 ± 13 Group B = 54 ± 13	25	38	2-weeks	MTS, FMA, BI, HAMD
Serag et al. (20)	RCT	MS	Group A = 7.9 ± 5 (weeks) Group B = 5.8 ±3.2 (weeks)	Group A = rPMS Group B = Sham rPMS	26	Group A = 18 Group B = 8	Group A = 34.6 ± 9.2 Group B = 32 ± 11.2	18	8	4-weeks	MAS, EDSS, 25 Foot Walking Test, Frequency and intensity of spasticity
Beaulieu et al. (24)	RCT	Stroke	Group A = 52.9 ± 36.7 (months) Group B = 82.7 ± 101.2 (months)	Group A = rPMS Group B = Sham rPMS	18	Group A = 9 Group B = 9	Group A = 51 ± 15 Group B = 55 ± 11	7	11	2-weeks	ROM, Isometric Muscle Strength, Resistance of plantar flexors to stretch, CME
Werner et al. (25)	RCT	Stroke TBI	Group A = 22.7 ± 8.8 (months) Group B =23.8 ± 6.4 (months)	Group A = rPMS + Sham rPMS + stretch Group B = Sham rPMS + rPMS + stretch	40	Group A = 20 Group B = 20	Group A = 47.9 ± 8.5 Group B = 55.4 ± 8.6	16	24	No	MAS, BI, Passive extension deficit
Chen et al. (19)	RCT	Stroke	Group A = 37.4 ± 42.0 (months) Group B = 45.6 ± 8.3 (months)	Group A = rPMS Group B = Sham rPMS	32	Group A = 16 Group B = 16	Group A = 49.0 ± 18.2 Group B = 45.6 ± 8.3	9	23	No	MAS, MTS, FMA, EEG
Jiang et al. (21)	RCT	Stroke	Group A = 13.81 ± 2.51 (days) Group B = 14.45 ± 3.33 (days)	Group A = rPMS + conventional physiotherapy Group B = conventional Physiotherapy	44	Group A = 24 Group B = 20	Group A = 54.62 ± 10.98 Group B = 56.09 ± 16.59	17	27	No	FMA, BI, MAS
Nahas et al. (26)	RCT	Stroke MS SCI Other	Group A = 42.74 ± 52.74 (months) Group B = 64.09 ± 67.07 (months)	Group A = rPMS Group B = Sham rPMS	36	Group A = 25 Group B = 11	Group A = 47.88 ± 14.8 Group B =41.60 ± 14.9	9	27	No	MAS, eBTD

AS, Ashworth Scale, PNF, Proprioceptive Neuromuscular Facilitation, EEG, electroencephalography, EMG, electromyogram, MS, multiple sclerosis, TBI, traumatic brain injury, SCI, spinal cord injury, rPMS, repetitive peripheral magnetic stimulation, MAS, Modified Ashworth Score, CME, corticomotor excitability, MTS, Modified Tardieu Scale, ROM, range of motion, FMA, Fugl-Meyer Assessment, BI, Barthel Index, HAMD, Hamilton depression scale, N/A, not available, EDSS, expanded disability status scale, eBTD, estimated botulinum toxin dose.

**Table 2:** Characteristics of rPMS parameters.

**References**	**Instrument**	**Frequency**	**Intensity**	**Duty cycle (OFF: ON)**	**Treatment time (min/session, sessions/w, w)**	**Total pulses**	**Stimulation Site**	**Coil type**
Nielsen et al. (22)	N/A	25 Hz	N/A	22:8	25 min/session, 7 sessions/w, 2 w	10,000	Eighth thoracic vertebra	N/A
Krewer et al. (23)	P-Stim 160 magnetic stimulator	25 Hz	10% of the muscle contraction threshold	2:1	20 min/session, 10 sessions/w, 2 w	5,000	Extensors and flexors of the upper and lower arm	Figure-of-eight coil
Serag et al. (20)	Dantec-Maglite magnetic stimulator	1 Hz	A fixed intensity of 45%	N/A	3 sessions/w,2 w	N/A	L2-4 spinal roots, 2 cm from midline	Figure-of-eight coil
Beaulieu et al. (24)	Magstim rapid^2^ Device	iTBS (3 @ 50 Hz delivered at 5 Hz)	42% of the maximal stimulator output	8:2	3.16 min/session	600	Tibialis anterior muscle belly	Figure-of-eight coil
Werner et al. (25)	Magstim rapid^2^ device	5 Hz	60%	3:3	5 min/ session	750	Forearm flexor muscles	Round coil
Chen et al. (19)	Mag-Pro R30 magnetic device	20 Hz: antagonistic muscle, 5 Hz: spastic muscle	100% of the muscle contraction threshold at a resting state	1:3 (5 Hz), 1:1.5 (20 Hz)	30 min/session	750 (5 Hz), 5,100 (20 Hz)	Upper limp	Round coil
Jiang et al. (21)	N/A	20 Hz	15% to 30% of the maximum instrument output.	2:0.5	20 min/s, 7 sessions/w, 2 w	2,400	Belly of paretic triceps brachii and extensor digitorum	Round coil
Nahas et al. (26)	Magnetic Magpro X100 Stimulator	iTBS (3@50 Hz delivered at 5 Hz)	Supra threshold intensity	8:2	3.33 min/session, 8 sessions	600	Lower limp	Figure-of-eight coil

w, week, N/A, not available.

**Table 3:** Aim, main results, and conclusions of included studies for this systematic review.

**References**	**Symptoms**	**Aim**	**Inclusion criteria**	**Exclusion criteria**	**Main results**	**Conclusions**	**Adverse events**
Nielsen et al. (22)	Severity of lower limb spasticity	To explore whether rPMS can improve spasticity in patients with MS.	(1) Clinical definite MS (2) Neurological condition for at least 6 months (3) Severity of lower limb spasticity (4) Preserved walking performance for 10 m	(1) Epilepsy, other neurological disorders, pregnancy and implanted spinal metal, drug infusion pump and pacemakers (2) Received magnetic stimulation previously	AS↓, EMG-, Self-Ease Score-	rPMS has an antispastic effect in MS.	No side effects
Krewer et al. (23)	Severe hemiparesis and mild to moderate spasticity	To investigate effects of rPMS on spasticity and motor function.	(1) Hemiparesis caused by stroke or TBI (2) Spasticity of an upper extremity with a score of 1–3 on the MTS (3) Ages between 18 and 75 years	(1) Metal implant in the head or within the stimulation area (2) Pregnancy and cardiac pacemaker, cochlea implant, or medication pumps (4) Comorbidity with neurodegenerative or orthopedic disorders (5) Increased intracranial pressure (6) Unstable fractures of the paretic upper extremity	MTS↓, MAS-, HAMD-	rPMS increase sensory function in patients with severe limb paresis in patients with CNS lesion. It has limited effect on spasticity and no effect on motor function.	No side effects
Serag et al. (20)	Spasticity and painful cramps in the lower extremities	To test the effectiveness of rPMS in decreasing spasticity and painful cramps in the lower extremities of patients with MS.	(1) MS diagnosis was made according to McDonald's criteria 2010 (2) EDSS score was less than or equal 6.5 (3) Spasticity grade 1+, 2, or 3 according to MAS (4) Refractory to oral medications for at least 3 months	(1) Fixed contractures were excluded as well as pregnant ladies (2) Implanted pacemakers or metallic devices	MAS↓, 25 Foot Walking Test-, Frequency and intensity of spasticity↓	rPMS has an antispastic effect in MS.	No side effects
Beaulieu et al. (24)	Chronic stroke patients with ankle impairments.	To explore whether rPMS could mediate improvements in corticomotor and clinical outcomes associated with ankle impairments in chronic stroke.	(1) Participants with stroke presented with paretic ankle muscles with spasticity (2) CT or MRI scan taken within the last 5 years (3) Walk independently (i.e., no physical assistance) more than 10 m with or without an assistive device	(1) The use of anti-spastic medication (2) Past vertebral surgery, major circulatory, respiratory or cardiac disease, neurological disease/deficit other than stroke (3) Severe lower limb orthopedic conditions, or cognitive disorder	ROM↑, CME↑, Strengthen↑, plantar flexor resistance to high-speed stretch↓	rPMS improved ankle impairments in chronic stroke patients.	No side effects
Werner et al. (25)	Chronic patients after CNS lesion with a severe wrist and finger flexor spasticity	To assess the effect of a single session of rPMS combined with manual stretch on wrist and finger flexor muscle spasticity.	(1) Patients with a single history of CNS lesion due to stroke or traumatic brain injury (2) Lesion interval >12 months (3) Increased muscle tone, Ashworth Score (0–5) in wrist or finger joints	(1) Volitional distal motor function of the affected arm, except for mass flexion (2) Metal implants or /and open wounds in the stimulation area (3) Deep vein thrombosis (4) Relevant edema (5) Pacemaker (6) Preceding BTX injection within the last 6 months	MAS↓, Passive Extension Deficit↓	A single session of rPMS combination with manual stretch significantly reduced the wrist and finger flexor muscle spasticity in patients with CNS lesion.	No side effects
Chen et al. (19)	Spasticity (MAS ≥ 1)	To explore the EEG mu rhythm change and decrease in spasticity after rPMS intervention in patients with stroke.	(1) Ischemic or hemorrhagic stroke diagnosed through computed tomography or MRI (2) Age in the range of 18–80 years (3) At least 2 weeks since stroke onset (4) Spasticity (MAS ≥ 1) (5) Ability to sit on a chair independently for at least 1 h	(1) Cardiac pacemaker (2) Pregnancy (3) Allergy to EEG electrode cream (4) Joint contracture in the hand or upper limb (5) Unstable fracture in the paretic upper limb	MAS↓, MTS↓, FMA↑	rPMS can reduce spasticity	No side effects
Jiang et al. (21)	No practical arm function within four weeks of a first stroke.	To investigate the effect of rPMS applied in early subacute stroke on severe upper extremity impairment.	(1) First-ever unilateral ischemic or hemorrhagic stroke in the basal ganglia with a course of 1–4 weeks (2) Medically stable (3) Age 30–80 years (4) A Brunnstrom stage of 1 to 2 for the upper limb and hand (5) Ability to provide written informed consent	(1) Severe spasticity (MTS>3) (2) Severe aphasia or cognitive impairment (3) Infection near the stimulation site (4) Deep-vein thrombosis near the stimulation site (5) Unstable fractures of the paretic upper extremity (6) Any contraindications to rPMS (e.g., metal implants in the affected limb or use of a pacemaker) (7) BTX injection, anti-spastic medicine	FMA↑, BI ↑	rPMS can improve arm function and muscle strength for grip and elbow flexion and extension.	No side effects
Nahas et al. (26)	Limb spasticity secondary to various neurological disorders	To investigate whether piTBS will reduce spasticity when applied directly on spastic muscles.	(1) Age more than 18 years, disease duration>6 months with persistent spasticity in the affected muscle and no change in anti-spasticity medications for at least 1 month prior to recruitment	(1) Recent BTX injection (< 4 months) (2) Metal plates, pacemakers, pregnancy	MAS↓, eBTD↓	piTBS could be a promising method to reduce spasticity and BTX in patients with CNS lesion.	No side effects

BI, Barthel Index, BTX, Botulinum toxin, CNS, central nervous system, CT, computerized tomography, EEG, electroencephalography, FMA, Fugl–Meyer Assessment, MAS, Modified Ashworth Score, MS, multiple sclerosis, MTS, Modified Tardieu Scale, MRI, magnetic resonance imaging, piTBS, peripheral intermittent theta burst stimulation, ROM, range of motion, CME, corticomotor excitability, rPMS, repetitive peripheral magnetic stimulation, TBI, traumatic brain injury, eBTD, estimated Botulinum toxin dose.

### Participants

A total of 297 patients with spastic paralysis were enrolled in eight RCTs, including 170 patients with stroke, 68 patients with MS, 50 patients with TBI, 5 patients with SCI, and 4 patients with other neurological disorders. There were 164 patients in the experimental group and 133 patients in the control group, with more male patients than female patients (127 females and 170 males). The patients' spasticity condition lasted longer than 6 months, and their ages ranged from 18 years to 80 years.

### Interventions

The number of sessions of rPMS in all included studies ranged from 1 to 20. Three studies (19, 24, 25) used single session, whereas the other five studies (20–23, 26) adopted multiple treatments with 6 to 20 sessions delivered in 8 days to 2 weeks. Five studies (19, 20, 22, 24, 26) directly compared rPMS with sham stimulation, and three (21, 23, 25) assessed the effects of rPMS plus conventional treatment including passive stretch (25), proprioceptive neuromuscular facilitation (21, 23), and neuromuscular electrical for spasticity.

For comparators, five studies adopted sham rPMS (19, 20, 22, 24, 26), two studies (23, 25) used sham rPMS combined with conventional treatment, and only one study (21) applied conventional physiotherapy alone. To achieve sham stimulation, the magnetic stimulators were connected with inactive coils in four studies (19, 23, 25, 26). The other protocols for sham rPMS involved using of obstacles between coil and limb (22), vertical coil placement (20) and very low intensity to non-target body part (24).

Among the eight studies, only two studies used iTBS pattern to stimulate spastic muscles of the lower limb (24, 26), whereas other studies adopted a routine repetitive pattern. Chen et al. adopted 20 Hz to stimulate antagonistic muscles and 5 Hz to stimulate spastic muscles. Frequencies at 20 and 25 Hz were used in four studies (19, 21–23), whereas Serag et al. used 1 and 5 Hz, respectively. In terms of coil type selection, four studies used a figure-eight coil (20, 21, 23, 24), three studies used a round coil (19, 21, 25), and one study did not mention it (22).

As far as stimulated position was concerned, the stimulation coil was applied over the lumbar of patients with MS in two studies (20, 22). Magnetic field was used to stimulate extensors or flexors of the upper limb in four studies (19, 21, 23, 25), and two studies used rPMS to stimulate the lower limb (24, 26). Each study used various inter train periods for spasticity rehabilitation. The train of pulses lasted from 0.5 to 8 s, whereas the intermittent time lasted from 1 to 22 s. The ratio of OFF/ON of rPMS ranged from 1 to 4 in the included studies. Moreover, the number of total pulses ranged from 600 to 10,000. Four studies (20, 21, 24, 25) reported the stimulation intensity ranged from 15 to 60% of the maximum stimulator output, whereas three studies (19, 23, 26) adopted suprathreshold intensity to produce muscle contraction. One study (22) did not mention it.

### Outcome measures

The eight studies included a variety of outcome measures covering pain, spasticity, motor function, psychologic conditions, and ADL, details of which are presented in Table 1. In the evaluation of the degree of spasticity, Ashworth Scale (AS) (22), modified Ashworth Scale (MAS) (19, 20, 25, 26), and modified Tardieu Scale (MTS) (19, 23) were used commonly in the included studies. Beaulieu et al. performed three clinical tests (range of dorsiflexion, isometric muscle strength, and resistance of plantar flexors to stretch) to quantify the degree of ankle spasticity in patients with stroke (24). Werner et al. used goniometers to assess passive extension deficit to the neutral position of the wrist and metacarpophalangeal II-V joints (MCP) (25). For motor function, FMA was used to assess the upper limb in patients following CNS lesion in three studies (19, 21, 23), whereas Serag et al. used the 25 foot walking test to evaluate the lower limb function (20). ADL and psychological conditions were assessed using BI (21, 23, 25) and Hamilton Depression Scale (HAMD) (23), respectively.

Moreover, some neuroelectrophysiological outcomes were used to record relevant indicators in three included studies (19, 22, 24). Chen et al. (2020) adopted EEG to observed mu rhythm changes associated with decreased spasticity in patients with stroke. Nielsen et al. (22) used EMG to record the stretch reflex and the maximum H-reflex of the soleus muscle for spasticity variation in patients with MS. In the study of Beaulieu et al. (24), they examined the change in transcranial magnetic stimulation-induced corticomotor excitability in terms of amplitude and latency of motor evoked potential, silent period, short-interval intracortical inhibition, and facilitation.

### Effectiveness

The included RCTs reported that rPMS alone or in combination with other rehabilitation treatment has a positive influence on spasticity (19, 20, 22–26), motor function (19, 21), and ADL (21, 25). Moreover, ankle function, wrist mobility, and MCP joint mobility can be improved in association with spasticity reduction in patients after CNS lesion (24, 25). The meta-analysis for the effectiveness of rPMS on outcome measures with adequate data is presented below.

#### Spasticity

**Figure 4:**
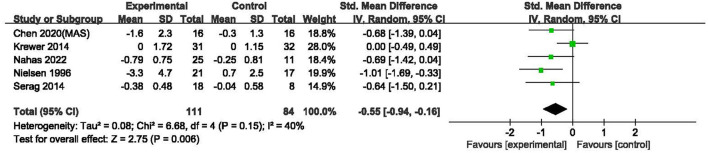
Meta-analysis of the effect of rPMS on spasticity measured with Ashworth scale, modified Ashworth scale, and modified Tardieu scale.

As the data of one study (25) could not be obtained, the cumulative effects of rPMS on spasticity assessed by the AS (22), MAS (19, 20, 26) and MTS (23) in five studies with 195 participants were analyzed in a SMD meta-analysis. Compared with control group, rPMS had a significant effect on spasticity reduction (SMD = −0.55, 95% CI: −0.94 to −0.16, *I*^2^ = 40%, and *P* = 0.006, Figure 4). It was noted that both MAS and MTS were assessed in Chen's study (2020). The data of MAS rather than MTS was put into this meta-analysis was because that MAS was more commonly to be used in clinical practice (27). On the other hand, if the MAS is replaced by MTS for Chen's study (2020), the results of this SMD meta-analysis would not be significantly changed with the cumulative effects size at –0.49 (95% CI: −0.87 to −0.11, *I*^2^ = 38%, and *P* = 0.01).

**Figure 5:**
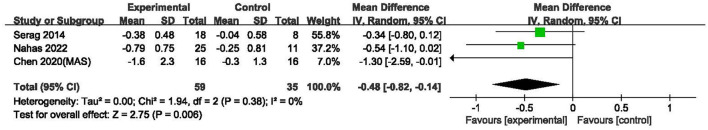
Meta-analysis of the effect of rPMS on spasticity measured with modified Ashworth scale.

Furthermore, a MD meta-analysis only for MAS in three studies involved 94 subjects (19, 20, 26) was performed. Compared with sham stimulation, rPMS had a significant effect on spasticity reduction (MD = −0.48, 95% CI: −0.82 to −0.14, *I*^2^ = 0%, and *P* = 0.006, Figure 5).

#### Motor function

**Figure 6:**
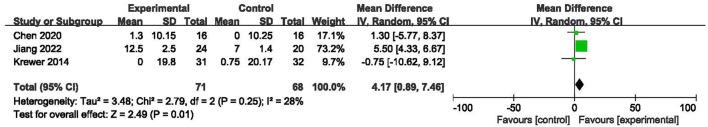
Meta-analysis of the effect of rPMS on motor function measured with Fugl-Meyer Assessment.

Three studies (19, 21, 23) including 139 subjects evaluated the effect of rPMS on motor function using the FMA scale. The *I*^2^-value for these studies was 28%. The results indicated a significant difference in change in FMA values between the two groups (MD = 4.17, 95% CI: 0.89 to 7.46, *I*^2^ = 28%, and *P* < 0.001, Figure 6). This result implied that rPMS improved motor function in individuals with spastic paralysis compared with the control group.

#### Activities of daily living

**Figure 7:**
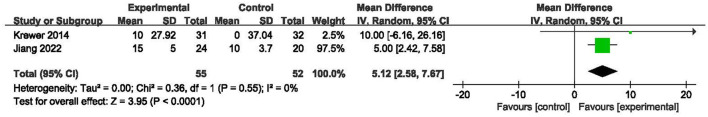
Meta-analysis of the effect of rPMS on activities of daily living measured with Barthel Index.

Two RCTs (21, 23) including 107 subjects investigated the effects of rPMS on ADL using the BI. The two studies adopted rPMS or sham stimulation plus occupational therapy or physiotherapy in the experimental and control groups. The results are shown in Figure 7. The homogeneities of the included studies were equal to 0%, Compared with the control treatment, rPMS had a significant effect on ADL (MD = 5.12, 95% CI: 2.58 to 7.67, *I*^2^ = 0%, and *P* < 0.0001, Figure 7), which indicated that rPMS could improve the ADL in individuals with spastic paralysis.

### Adverse effects

No adverse events were reported in any of the selected studies.

## Discussion

### The effects of rPMS

Spasticity is commonly treated with rPMS in clinical rehabilitation, but the existing evidence is limited in support of its validity. We collected eight RCTs to conduct this systematic review and meta-analysis on the effects of rPMS on the spasticity in patients with CNS lesions. Compared with sham stimulation, rPMS showed a significant reduction in spasticity and improved the motor function and ADL for patients with spastic paralysis.

In this review, three studies (19, 20, 26) involving meta-analysis assessed spasticity by using the MAS and one study (22) used AS. The results were consistent for rPMS in reducing spasticity compared with sham stimulation. In addition, two studies assessed the antispastic effects of rPMS with MTS. Krewer et al. (23) observed a reduction of spasticity in MTS at short term (wrist flexor) and long term (elbow extensor) when rPMS was combined with occupational therapy in patients with brain lesions. Chen et al. (19) also reported that rPMS significantly reduced spasticity in motion during the MTS test in patients following stroke. However, given that the protocols of MTS used in these two studies differed (19, 23), a meta-analysis specific to MTS could not be conducted. Hence, two meta-analysis on the effects of rPMS for all available outcomes (MAS, AS and MTS) and MAS alone were performed, respectively. The cumulative results demonstrated statistically positive effects of rPMS on spasticity reduction in patients with CNS lesion.

Previous systematic reviews (28, 29) have demonstrated a lack of sufficient evidence to support the effects of rPMS on motor function in patients with stroke. In this meta-analysis, however, the cumulative results showed the favorable effects of rPMS on motor function of the paretic limb in FAM score in patients with CNS diseases compared with the control group. Three studies showed inconsistent results. Two studies (19, 21) reported a significant improvement in FMA score after applying one session or multiple sessions of rPMS on upper and lower limbs alone or combined with conventional physical therapy in stroke patients. Another study (23) reported no change in arm motor function assessed by FMA after 2 weeks of rPMS prior to occupational therapy in patients following stroke and TBI. The authors supposed that the negative results on motor function might be due to the relatively short intervention period of rPMS. With BI as the outcome measure, two studies (21, 23) evaluated the effects of rPMS on ADL in patients with brain lesion. Jiang et al. (21) demonstrated that ADL significantly improved after 14 sessions of rPMS combined with conventional physical therapy compared with conventional physical therapy alone in stroke patients, whereas Krewer et al. (23) reported no significant improvement in BI after 20 sessions of rPMS combined with occupational therapy compared with the control group with sham rPMS plus occupational therapy.

The significant effect of meta-analyses indicates some benefit of the use of rPMS. The clinical importance of rPMS using for different function aspect should be taken seriously. For spasticity measured with MAS, Chen et al. (30) demonstrated that the minimal clinical important difference (MCID) were 0.48 and 0.76 for moderate and large effect size in upper extremity muscles, and 0.45 and 0.73 in lower extremity muscles, respectively. Hence, the effect size of 0.48 obtained from our meta-analysis on MAS may indicate a moderate clinically meaningful change in spasticity improvement from rPMS in patients with CNS lesion. Furthermore, the MCID values were suggested to be 5.25 for FMA (31) and 1.85 to 6.84 for BI (32, 33) in patients with brain lesion. In contrast, the effect size of our meta-analyses for FMA and BI were 4.17 and 5.12, respectively. Therefore, more solid evidence is required to support the clinical effects of rPMS on motor function and ADL in UMNL patients.

### The mechanism of rPMS on spasticity decrease

Spasticity is a symptom of hyperexcitability of the stretch reflex following UMNL (34). It is not only a motor disorder but also influenced by cutaneous and proprioceptive afferents (2). With early stroke patients, Wissel et al. (35) demonstrated that sensory deficit is one of the key risk factors associated with spasticity development. When rPMS is applied to the peripheral limbs, an increased somatosensory and proprioceptive afferent would be induced *via* a direct activation of sensorimotor nerve fibers with an orthodromic and antidromic conduction and an indirect activation of mechanoreceptors during rhythmic contraction-relaxation and muscle vibration (23, 29, 36). This increased proprioception and somatosensation might be beneficial in reducing spasticity. Meanwhile, the stretch reflex could be modulated by the higher centers in the motor pathway (2). Such afferent signals induced by rPMS input to the primary sensory cortex (S1) along the ascending sensory pathway might then up-regulate corticomotor excitability through structural and functional connections between sensory and motor cortices (13, 37). This cortical activation might lead to an increase in the inhibitory regulation of the stretch reflex, thereby reducing spasticity.

The other potential mechanism of rPMS on spasticity might relate to its local effect on the tissues. Okudera et al. (38) demonstrated a significant decrease in muscle hardness and increase in cephalic venous blood flow of extensor digitorum muscle measured *via* shear wave imaging after 600 magnetic pulses were delivered at 20 Hz to the radial nerve of the non-dominant hand in healthy subjects. Such effects were sustained for at least 15 min. This tissue-softening effect of rPMS may contribute to the reduced spasticity.

### Limitations

The interpretation of the results of this study should be confined to some limitations. First, the participants of included studies were designed as having spastic paralysis, however, the conditions are heterogeneous in spasticity severity. Thus, the spasticity reduction effects of rPMS could not be specific to the degree of spasticity. Secondly, although the outcome measurements for spasticity included in the present meta-analysis, namely AS, MAS and MTS, were commonly adopted to assess muscle tone in clinical practice, the domain of reflex mediated stiffness was not well-addressed. Therefore, future studies would benefit from the use of a more reliable and reproducible spasticity test capable of distinguishing passive muscle properties from reflex-mediated stiffness. Furthermore, all the meta-analyses in the present review are based on a small sample size (2–5 studies). It is not likely to obtain enough power to confirm the effect size for each outcome (39). Therefore, the level of evidence obtained was not robust. Finally, because of the varying number of sessions and stimulus duration involved in different included studies, further studies should be analyzed with the same rPMS parameters if the studies are sufficient.

### Clinical application and prospect

At present, evidence shows that rPMS is a promising intervention method for spasticity and motor function impairment due to CNS lesion. However, the protocol of rPMS (e.g., frequency, intensity, coil, number of pluses, and duty circle) is inconsistent. Thus, high-quality studies with a large sample size are necessary to confirm the optimal protocol of rPMS for clinical practice in spasticity treatment.

## Conclusion

Results revealed that rPMS had the potential effects of reducing spasticity especially for passive muscle properties evaluated with AS/MAS, and improving motor function and ADL in patients with spastic paralysis. Future studies are encouraged to design high-quality trials that include more patients and incorporate standard outcome measurement to explore the optimal protocol of rPMS in patients with spastic paralysis.

## Data availability statement

The original contributions presented in the study are included in the article/Supplementary material, further inquiries can be directed to the corresponding author/s.

## Author contributions

HL, J-XP, and Y-BJ designed the structure and scope of the review. J-XP and Y-XD searched and reviewed the literature and drafted the manuscript. M-YW and Y-LW provided statistical support. HL, Y-BJ, H-YP, X-ZW, and L-RL revised the manuscript. All authors approved the final version of the manuscript.

## Funding

This work was supported by the Science and Technology Innovation Program (Social Development) in Yixing (Grant Number 2021SF21) and the National Key Research & Development Program of Ministry of Science and the Technology of the People's Republic of China (Grant Numbers 2020YFC2006100 and 2020YFC2006104).

## Conflict of interest

The authors declare that the research was conducted in the absence of any commercial or financial relationships that could be construed as a potential conflict of interest.

## Publisher's note

All claims expressed in this article are solely those of the authors and do not necessarily represent those of their affiliated organizations, or those of the publisher, the editors and the reviewers. Any product that may be evaluated in this article, or claim that may be made by its manufacturer, is not guaranteed or endorsed by the publisher.
