# Supplementary material for: Effects of repetitive peripheral magnetic stimulation on spasticity evaluated with modified Ashworth scale/Ashworth scale in patients with spastic paralysis: A systematic review and meta-analysis
Source: Front Neurol. 2022 Nov 8;13:997913. doi: 10.3389/fneur.2022.997913 (PMC9679494; doi:10.3389/fneur.2022.997913)
Supplement: Supplementary file 1 [file Data_Sheet_1.docx]

**Appendix**

Search strategy

PubMed:

1. Spasticity[Mesh] OR Spasticity, Muscle[Title/Abstract] OR Spastic[Title/Abstract] OR Clasp-Knife Spasticity[Title/Abstract] OR Clasp Knife Spasticity[Title/Abstract] OR Spasticity, Clasp-Knife[Title/Abstract]
2. Spasms[Mesh] OR Muscle Spasm[Title/Abstract] OR Muscle Spasms [Title/Abstract] OR Spasm, Muscle[Title/Abstract] OR Spasms, Muscle[Title/Abstract] OR Muscular Spasm[Title/Abstract] OR Muscular Spasms[Title/Abstract] OR Spasm, Muscular[Title/Abstract] OR Spasms, Muscular[Title/Abstract] OR Spasm, Generalized[Title/Abstract] OR Generalized Spasm[Title/Abstract] OR Generalized Spasms[Title/Abstract] OR Spasms, Generalized[Title/Abstract] OR Spasm, Ciliary Body[Title/Abstract] OR Ciliary Body Spasm[Title/Abstract] OR Ciliary Body Spasms[Title/Abstract] OR Spasms, Ciliary Body[Title/Abstract]
3. Muscular tension[Mesh] OR Muscular tone[Title/Abstract] OR Tonus, Muscle[Title/Abstract] OR Muscle Tightness[Title/Abstract] OR Tightness, Muscle[Title/Abstract] OR Muscular Tension[Title/Abstract] OR Tension, Muscular[Title/Abstract] OR Muscle Tension[Title/Abstract] OR Tension, Muscle[Title/Abstract] OR
4. Dystonia[Mesh] OR Muscle Dystonia[Title/Abstract] OR Dystonia, Muscle[Title/Abstract] OR Dystonia, Paroxysmal[Title/Abstract] OR Paroxysmal Dystonia[Title/Abstract] OR Dystonia, Diurnal[Title/Abstract] OR Diurnal Dystonia[Title/Abstract] OR Dystonia, Limb[Title/Abstract] OR Limb Dystonia[Title/Abstract]
5. Muscle Hypertonia[Mesh] OR Hypertonia, Muscle[Title/Abstract] OR Hypertonias, Muscle[Title/Abstract] OR Muscle Hypertonias[Title/Abstract] OR Muscle Tone Increased[Title/Abstract] OR Increased, Muscle Tone[Title/Abstract] OR Tone Increased, Muscle[Title/Abstract] OR Muscular Hypertonicity[Title/Abstract] OR Hypertonicities, Muscular[Title/Abstract] OR Hypertonicity, Muscular[Title/Abstract] OR Muscular Hypertonicities[Title/Abstract] OR Hypermyotonia[Title/Abstract] OR Hypermyotonias[Title/Abstract] OR Hypertonia, Transient[Title/Abstract] OR Hypertonias, Transient[Title/Abstract] OR Transient Hypertonia[Title/Abstract] OR Transient Hypertonias[Title/Abstract] OR Hypertonia, Neonatal[Title/Abstract] OR Hypertonias, Neonatal[Title/Abstract] OR Neonatal Hypertonia[Title/Abstract] OR Neonatal Hypertonias[Title/Abstract] OR Hypertonia, Sphincter[Title/Abstract] OR Hypertonias, Sphincter[Title/Abstract] OR Sphincter Hypertonia[Title/Abstract] OR Sphincter Hypertonias[Title/Abstract] OR Hypertonia, Detrusor Muscle[Title/Abstract] OR Detrusor Muscle Hypertonia[Title/Abstract] OR Detrusor Muscle Hypertonias[Title/Abstract] OR Hypertonias, Detrusor Muscle[Title/Abstract] OR Muscle Hypertonia, Detrusor[Title/Abstract] OR Muscle Hypertonias, Detrusor[Title/Abstract] OR Hypertonia, Infantile[Title/Abstract] OR Hypertonias, Infantile[Title/Abstract] OR Infantile Hypertonia[Title/Abstract] OR Infantile Hypertonias[Title/Abstract] OR
6. #1 OR #2 OR #3 OR #4 OR #5
7. (RPMS [Title/Abstract] OR "repetitive peripheral magnetic stimulation" [Title/Abstract] OR "functional magnetic stimulation" [Title/Abstract] OR "transcutaneous magnetic stimulation" [Title/Abstract] OR "peripheral magnetic stimulation" [Title/Abstract] OR magnetic stimulation [Title/Abstract])
8. (clinical[tiab] AND trial[tiab]) OR "clinical trials as topic"[mesh] OR "clinical trial"[pt] OR random*[tiab] OR "random allocation"[mesh] OR "therapeutic use"[sh]
9. #6 AND #7 AND #8

Cochrane Library:

#1 MeSH descriptor: [Muscle Spasticity] explode all trees

#2 MeSH descriptor: [Spasm] explode all trees

#3 MeSH descriptor: [Muscle Tonus] explode all trees

#4 MeSH descriptor: [Dystonia] explode all trees

#5 MeSH descriptor: [Muscle Hypertonia] explode all trees

#6 #1 OR #2 OR #3 OR #4 OR #5 in Trials

#7 (RPMS):ti,ab,kw

#8 (repetitive peripheral magnetic stimulation):ti,ab,kw

#9 (magnetic stimulation):ti,ab,kw

#10 (functional magnetic stimulation):ti,ab,kw

#11 (transcutaneous magnetic stimulation):ti,ab,kw

#12 (peripheral magnetic stimulation):ti,ab,kw

#13 #7 OR #8 OR #9 OR #10 OR #11 OR #12 in Trials

#14 #6 AND #13

Web of Science:

1. TS=(Spasticity OR Spasticity, Muscle OR Spastic OR Clasp-Knife Spasticity OR Clasp Knife Spasticity OR Spasticity, Clasp-Knife OR Spasms OR Muscle Spasm OR Muscle Spasms OR Spasm, Muscle OR Spasms, Muscle OR Muscular Spasm OR Muscular Spasms OR Spasm, Muscular OR Spasms, Muscular OR Spasm, Generalized OR Generalized Spasm OR Generalized Spasms OR Spasms, Generalized OR Spasm, Ciliary Body OR Ciliary Body Spasm OR Ciliary Body Spasms OR Spasms, Ciliary Body OR Quadriplegias OR Tetraplegia OR Tetraplegias OR Spastic Quadriplegia OR Quadriplegia, Spastic OR Quadriplegias, Spastic OR Spastic Quadriplegias OR Spastic Tetraplegia OR Spastic Tetraplegias OR Tetraplegia, Spastic OR Tetraplegias, Spastic OR Muscular tension OR Muscular tone OR Tonus, Muscle OR Muscle Tightness OR Tightness, Muscle OR Muscular Tension OR Tension, Muscular OR Muscle Tension OR Tension, Muscle OR Dystonia OR Muscle Dystonia OR Dystonia, Muscle OR Dystonia, Paroxysmal OR Paroxysmal Dystonia OR Dystonia, Diurnal OR Diurnal Dystonia OR Dystonia, Limb OR Limb Dystonia OR Muscle Hypertonia OR Hypertonia, Muscle OR Hypertonias, Muscle OR Muscle Hypertonias OR Muscle Tone Increased OR Increased, Muscle Tone OR Tone Increased, Muscle OR Muscular Hypertonicity OR Hypertonicities, Muscular OR Hypertonicity, Muscular OR Muscular Hypertonicities OR Hypermyotonia OR Hypermyotonias OR Hypertonia, Transient OR Hypertonias, Transient OR Transient Hypertonia OR Transient Hypertonias OR Hypertonia, Neonatal OR Hypertonias, Neonatal OR Neonatal Hypertonia OR Neonatal Hypertonias OR Hypertonia, Sphincter OR Hypertonias, Sphincter OR Sphincter Hypertonia OR Sphincter Hypertonias OR Hypertonia, Detrusor Muscle OR Detrusor Muscle Hypertonia OR Detrusor Muscle Hypertonias OR Hypertonias, Detrusor Muscle OR Muscle Hypertonia, Detrusor OR Muscle Hypertonias, Detrusor OR Hypertonia, Infantile OR Hypertonias, Infantile OR Infantile Hypertonia OR Infantile Hypertonias )
2. TS= (repetitive peripheral magnetic stimulation OR RPMS OR functional magnetic stimulation OR transcutaneous magnetic stimulation OR peripheral magnetic stimulation OR magnetic stimulation)
3. TS= (clinical trial OR random* OR random allocation OR random* controlled trial OR placebo)
4. #1 AND #2 AND #3

Embase:

1. 'spasticity'/exp OR 'muscle tone'/exp OR 'dystonia'/exp OR 'muscle hypertonia'/exp
2. ('spastic':ab,ti OR 'spasms':ab,ti OR 'muscle tension':ab,ti OR 'limb dystonia':ab,ti) AND 'muscle tone increased':ab,ti
3. #1 OR #2
4. 'rpms':ti,ab OR 'repetitive peripheral magnetic stimulation':ab,ti OR 'functional magnetic stimulation':ti,ab OR 'transcutaneous magnetic stimulation':ab,ti OR 'peripheral magnetic stimulation':ab,ti OR 'magnetic stimulation':ab,ti
5. 'clinical':ti,ab AND 'trial':ti,ab OR 'clinical trial'/exp OR random*
6. #3 AND #4 AND #5
